# Pregnant women’s perception on the health effects of household air pollution in Rural Butajira, Ethiopia: a phenomenological qualitative study

**DOI:** 10.1186/s12889-023-16578-8

**Published:** 2023-08-25

**Authors:** Sisay Shine, Mulugeta Tamirie, Abera Kumie, Adamu Addissie, Simon Athlin, Hussen Mekonnen, Eshetu Girma, Mitike Molla, Mirgissa Kaba

**Affiliations:** 1 Department of Public Health, School of Public Health, Debre Berhan University, P.O.Box 445, Debre Berhan, Ethiopia; 2 Department of Preventive Medicine, School of Public Health, Addis Ababa University, Addis Ababa, P.O. Box 9086, Ethiopia; 3 School of Medical Sciences, Örebro University, Örebro, Sweden

**Keywords:** Household air pollution, Pregnant mother perception, Qualitative study, Butajira, Ethiopia

## Abstract

**Background:**

Household air pollution is the major public health problem in developing countries. Pregnant women spent the majority of their time at home and are the most affected population by household air pollution. Exploring the perception of pregnant women on adverse health effects is important to enhance the mitigation strategies. Therefore, this study aim to explore the pregnant women’s perceptions about health effects of household air pollution in rural Butajira, Ethiopia.

**Methods:**

A phenomenological qualitative study design was conducted among 15 selected pregnant women. All interviews were carried out at the participants´ house and audio-recorded while housing and cooking conditions were observed and appropriate notes were taken for each. The collected data were transcribed verbatim and translated into the English language. Then, the data were imported into Open code software to manage the overall data coding processes and analyzed thematically.

**Results:**

Study participants perceived that respiratory problems such as coughing, sneezing and asthma and eye problem were the major health problem caused by household air pollution among pregnant women. Study participants also mentioned asphyxiated, abortion, reduces weight, and hydrocephalus was caused by household air pollution on the foetus. Study participants perceived that financial inability, spouse negligence, autonomy and knowledge level of the women were the barriers to tackling household air pollution. Study participant also suggested that opening the door and window; using improved cookstove and reduce workload were the perceived solution for household air pollution.

**Conclusions:**

This study explores pregnant women’s perceptions on health effects of household air pollution. The finding of this study was important to deliver suitable intervention strategies to mitigate household air pollution. Therefore, educating the women on way of mitigating household air pollution, improving existing structure of the house and minimize the time to stay in the kitchen is important to mitigate household air pollution exposure.

## Background

The use of biomass fuels for preparing food is the major source of household air pollution (HAP) on a global scale [1]. Nearly 50% of the world population continues to cook with biomass fuels such as dung, wood, and agricultural residues [2]. In low-income countries, more than 80% of rural communities and more than 95% of households in Ethiopia are dependent on biomass fuel for cooking their food [3, 4].

Globally, household air pollution is the cause for nearly 4 million premature deaths, including over.

400,000 deaths of children under 5 years and 85.5 million disability-adjusted life year (DALY) in 2016 [5, 6], more than 90% occurred in low and middle-income countries. Sub-Saharan African countries had the highest mortality rate (120/100,000 in 2019) in the world due to household air pollution [7, 8]. In Ethiopia, HAP was estimated to be responsible for more than 50,000 deaths and the cause of nearly 5% of the national burden of disease 2007 [9, 10].

Pregnant women are the most affected segment of the population due to spending the majority of their time is at home and the high rate of solid fuel utilization throughout their pregnancy period for the domestic and cooking role [5, 11]. The level of exposure for pregnant women can also be determined based on the presence of a window, kitchen, chimney, the construction materials of the house, how often the windows are opened, and the type of fuel used [12, 13]. Studies revealed that HAP exposure is the cause of asthma, eye irritation, tuberculosis, heart disease, lung disease, and nasopharyngeal cancers, and also causes an interrupted foetus development and escalates the risk of premature birth, stillbirth, and low birth weight [11, 14, 15].

The government of Ethiopia and other low-income countries have designed different strategies, e.g. the use of locally available improved cookstoves, to minimize HAP concentrations associated with biomass fuel at the household level [16]. A study conducted in Ethiopia indicated that more than 83% of rural household use traditional three clay stoves and HAP level was up to 27.3 times higher than the WHO 24-h guideline limits [17, 18]. Therefore, involving local pregnant women, the source of experiential knowledge and a primary target audience, to design interventions for HAP is crucial [18–20]. However, due to lack of giving emphasis on the field studies on pregnant women perception about the health effects of HAP are insufficient in Ethiopia. Therefore, the purpose of this study was to explore the perception of pregnant woman on the health effect of household air pollution.

## Methods

### Study setting

The study was conducted in rural Butajira woreda in February 2022. Butajira woreda is located in Gurage Zone of Southern Nations and Nationalities and Peoples regional state of Ethiopia, approximately 136 km south of Addis Ababa at a moderate altitude of 2,131 m. Butajira woreda has been the Health and Demographic Surveillance Site (HDSS) of Addis Ababa University Rural Health programme since 1986. The climate varies from dry lowland areas at altitudes around 1,500 m (tropical climate) to cool mountainous areas up to 3,500 m above mean sea level (temperate climate). Most houses in rural areas of Butajira are tukul type, round huts built from mud with thatched roofs. The total population in the woreda is approximately 85,799, of whom 44,143 (51.4%) are females [21, 22].

### Study design

In this study, we applied a phenomenological qualitative research study design to explore the pregnant women’s perception on the health effect of household air pollution in rural Butajira. This design would allow for an in-depth exploration of the individual experience and perception of the pregnant women regarding the health effect of household air pollution. Phenomenological design focuses on understanding the essence of the experience of a particular phenomenon, in this case, the experience of pregnant woman with regard to household air pollution. This design would involve interviewing pregnant woman individually to gather their personal experience and perception. It also used for studies which are descriptive in nature, particularly for examining healthcare-related phenomena and is identified as important and appropriate for research questions focused on discovering the ‘who, what and where’ of events or experience and gaining insights from informants regarding a poorly understood phenomenon. The data collected from these interviews would be analysed thematically to identify cohesions and differences in their experiences and perceptions [23, 24].

### Participants and recruitment

In this study, mixed purposive sampling using maximum variation and criterion sampling were employed to recruit the study participants. Maximum variation sampling was used to yield a wider perspective of the participants’ perception from different geographic areas of the woreda. The Woreda represents all the three lowland, midland, and highland agro-ecology sites of Ethiopia at which authors believed the perception of pregnant women on health effect of household air pollution is different. We selected a total of 15 study participants from three different geographic areas (lowland, midland and highland). Pregnant women who used biomass fuel for cooking and in their third trimester were recruited for the study. The level of household air pollution exposure among geographic areas (lowland, midland and highland) were different due to the utilization of biomass full differently and the perception of the pregnant women on household air pollution health effects were also different among geographic areas therefore, we recruited the participants across a different geographic areas [25].

### Data collection process

In-depth interview was conducted with the pregnant women in their home. The interviews were prescheduled to guarantee maximum privacy. The interview was continued until the saturation level of the data was reached. The principal investigator (SS) in consultation with (MT) collected the data in the field. Ten semi-structured interview guide with probing questions was used to get the required information from the study participants. The interview was conducted in Amharic, the regional working language the participants speak. The interview duration ranged from 40 to 60 min with the main themes of discussion focusing on the women’s perception on the health effects of household air pollution, perceived psychosocial effect, perceived barriers to tackling the exposure, and possible solutions to reduce household air pollution exposure. Short notes were maintained during the in-depth interviews and later expanded to field notes and a summary report to capture non-verbal and facial expressions during the interview. The field notes were reviewed and audio tapes listened to in order to follow the emerging themes, and check for data saturation point, modify the interview guide and determine the number of participants. The redundancy of ideas was visible after the 13th study participant however; two more interviews were conducted to confirm the saturation level was reached. All audio files and field notes of the interviews were fully transcribed in Amharic then translated into the English language.

### Trustworthiness

The quality of the study was maintained by the rigor criteria of credibility, dependability, conformability and transferability [26]. The credibility of the study was maintained by the specific activities. Discussion was held with the lead authors and the study participants to develop a trusting relationship; the lead author had attended an advanced qualitative research course and had frequent discussions with the supervisor reporting the preliminary methods and findings; the local co-ordinator introduced the participants to project. Prolonged time spent with participants and using the participants’ words in the final report was increase the credibility of the study. After completion of the data collection, reflection sessions were prepared with the principal investigator (SS) and supervisors (AK and MT). The dependability of the study was maintained by the transcribed data systematically reviewed against the audio files. The study participants were also probed to elaborate on or clarify relevant point raised. The translated data were coded by first year PhD students and the coding results discussed by the coders. Discrepancies in the interpretations were negotiated. Transferability of the data was maintained by a rich description of the method section of the study and representative participants were included from lowland, midland and highland areas of rural Butajira reflecting diverse groups in terms of indoor air pollution exposure. The study finding confirmability was guaranteed with the research supported by the data collected. Triangulated on the ground data were gathered with authenticity considering fairness to represent all views adequately. The interview was continued until the saturation levels of the data were reached. A meeting session was arranged each day to discuss the daily activities with the principal investigator (SS) and the supervisor for consultation (MT).

### Data management and analysis

The qualitative data from pregnant women interviews were analysed using thematic analysis by importing the transcribed text into the Open Code programme to facilitate the coding process. First, the PI and the researcher assistant read the complete transcripts and gave codes for descriptive categories, which were then developed inductively in order to develop themes which are related to the pregnant women’s understanding of perceived physical and psychological health effects of household air pollution, their solutions and barriers to realizing to take appropriate measures for prevention. Open code version 4.03 software was used to manage the overall data coding processes.

## Results

### Socio-demographic characteristics of study participants

A total of 15 in-depth interviews with pregnant women who were primary family cook, third trimesters, and lived rural Butajira were included. Ten of the study participants were above 25 years old, 12 were multi-parity and 10 attended formal education. Twelve of the houses in the study area were made of corrugated iron, 9 of the participants’ houses had windows in the living room and their kitchen is attached to the main house, 13 study participants shared their main house with animals (Table 1). The findings were structured into sections according to the seven themes and seventeen sub-themes. The main themes and sub-themes were, definition of HAP (smoke and bad odour); source of HAP (using biomass fuel as source energy, dust and vehicle); perceived health effects of HAP on pregnant women (respiratory and eye problem); perceived health effects of HAP on foetus (asphyxiated, abortion, reduce weight and hydrocephalus); perceived psychosocial effects of HAP (inferiority and depression); perceived barriers to tackling the exposure of HAP (financial, spouse negligence, autonomy and knowledge); and perceived solutions to prevent HAP (open the door and window, separate kitchen and ceiling height, use improved cookstove and reduce workload).

### Household air pollution definition

#### Smoke

Those who took part in the research explained household air pollution as smoke generated when cooking food inside homes using wood and animal waste. A 24-year-old pregnant woman, who resided in the mid-land area of the kebele, mentioned that: *“…yes, I can say that household air pollution is the suffocation of the house by the smoke …” (* *A 24-years old pregnant women, P10* *)*

#### Bad odour

Respondents also used the term “foul odour” to describe household air pollution, which resulted from multiple factors such as leftover food, discarded dishwashing water, and pets inhabiting the living room. A 28-year-old participant from the lowland kebele’s shared this perspective by mentioning like this: *“….household air pollution is the bad odour found in the house raised from different sources like cattle’s which we shared the same living room…” (* *A 28-years old pregnant women, P6* *).*

### Source of household air pollution

#### Using biomass fuel as source of energy

Study participants had different understanding about the source of air pollutants in their homes. All participants mentioned that household air pollutants were released from burning of wood, charcoal, animal dungs and crop residues during the preparation of food in the house. One of the participants from the highland area, who is illiterate in both reading and writing, stated this as follows: *“….as of my understanding the main source of household air pollution here in our kebele is using wood, crop residues and animal dung for the preparation of our daily food preparation…” (* *A 35-years old participant, P2* *).*

#### Dust

In addition, six individuals in the study held the belief that household air pollution is caused by dust generated from cleaning activities and dust that enters the house from various sources like the wind. One of the participants, who was 25 years old, expressed the following opinion: *“Our home’s air pollution may be mostly caused by the dust that rises from cleaning the house and the strong winds that frequently blow here in the lowland area, especially during the winter season.” (* *A 25-years old participant, P14* *).*

#### Vehicle emission

The participants also indicated that vehicle emissions are another source of household air pollution. Three participants indicated that the smoke emitted from the motor bike and Bajaj pollutes our home air, despite the fact that the study area is rural and vehicle access is uncommon. A 28-year-old pregnant woman with a grade 8 educational status described the scenario as follows: *“Yes, vehicle smoke pollutes the air inside the home, but since our area is rural, there aren’t many cars around. However, when Bajajs and motorbikes arrive in our area, the smoke they generate enters to our home and contaminates the air.” (* *A 28-years old participant, P13* *).*

**Table 1:** Background information of the study participants in rural Butajira, Ethiopia 2022

**ID**	Location	**Age**	**Parity**	**Education attended**	**House type**	**Kitchen constructed**	**Animal live in main house**	**Living room have window**
P1	Highland	23	2	Can’t read & write	Corrugated iron	Separated	No	Yes
P2		35	3	Can’t read & write	Corrugated iron	Attached	Yes	Yes
P3		27	3	Can’t read & write	Corrugated iron	Attached	Yes	No
P4		38	8	Grade 4	Corrugated iron	Attached	Yes	Yes
P5		28	5	Grade 6	Corrugated iron	Separated	No	Yes
P6	Midland	24	2	Grade 8	Corrugated iron	Attached	Yes	Yes
P7		26	3	Grade 8	Corrugated iron	Attached	Yes	Yes
P8		20	1	Grade 7	Corrugated iron	Attached	Yes	No
P9		28	6	Can’t read & write	Tukul	Attached	Yes	No
P10		24	1	Grade 7	Corrugated iron	Attached	Yes	Yes
P11	Lowland	28	4	Grade 4	Tukul	Attached	Yes	No
P12		35	6	Can’t read & write	Corrugated iron	Attached	Yes	Yes
P13		28	4	Grade 8	Tukul	Attached	Yes	No
P14		25	4	Grade 9	Corrugated iron	Separated	No	Yes
P15		28	3	Grade 10	Corrugated iron	Attached	Yes	No

### Perceived health effects of HAP on pregnant women

#### Respiratory problem

A number of medical disorders appear to be brought by HAP. The women had various opinions about the harm that HAP did to their personal health. The effects of air pollution on a person’s health can range from mild breathing difficulties to severe eye problem. Thirteen participants mentioned that HAP was the root cause of coughing, sneezing and asphyxiated. One of the participant mentioned this like; *“If we cook in the living room using charcoal and animal dung without full combustion, it would cause serious health issues, especially for our respiratory system, causing asthma, coughing, and sneezing.” (* *A 23-years old participant, P1* *)*.

#### Eye problem

Human eyes are negatively affected by household air pollutants mostly through irritation and inflammation, with conjunctivitis being a common issue. The causes of itching and eyesight were similarly mentioned by more than half of the study’s participants as being related to household air pollution. For instance one of the study participants mentioned this as follows: *“… my eyes tend to itch and tear easily when my home is filled with smoke, which can eventually cause blindness.” (* *A 28-years old participant, P9* *)*.

### Perceived health effects of HAP on the foetus

#### Asphyxiated

Participants believed that household air pollution during pregnancy is the cause for deprivation of oxygen to the foetus. Five of the study participants mentioned that household air pollution could cause asphyxiated the foetus. For instance a 38 years old pregnant woman mentioned this as follows: “*… when there is high smoke level in the house and exposed for that smoke the foetus becomes asphyxiated.*” *(* *A 38-years old participant, P4* *)*.

#### Abortion

Majority of the pregnant women believed that exposure to the household air pollution could cause abortion of the foetus. Participants’ high levels of indoor smoke exposure were said to have caused abortions of the foetus. A pregnant woman participant, who can’t read and write in their educational status, said that: *“Yes, I had an abortion during my first pregnancy, which I believe may have been caused by the high levels of smoking in the house at the time.” (* *A 35-years old participant, P2* *)*.

#### Reduced weight

Household air pollution is related with an increased risk of low birth weight of foetus. Most participants described that household air pollution during pregnancy could lead to reduce the birth weight of the foetus. “*I think household air pollution can affect our foetus in different ways for instance it could reduce the weight of the foetus/the foetus became thin.” (* *A 28-years old participant, P5* *)*.

#### Hydrocephalic

Abnormal build-up of cerebrospinal fluid in the brain’s ventricle is another health problem faced on the foetus. Participants also believed that household air pollution is cause for hydrocephalus. One of the study participants told her story about the impact of household air pollution on the foetus. *“…. I had an experience of seventh-month foetus termination in the hospital because of the doctor told me that foetus accumulated water in his head (hydrocephalus). When I ask myself why this happen? I believed that it may be due to the problem of household air pollution exposure in my house….”* *(A 29-years-old participant, P9)*.

### Perceived psychosocial effect HAP on the women

#### Feeling inferiority

Pregnant women perceived household air pollution has different psychosocial impacts on them. They thought that having a high quantity of smoking in their home made them feel inferior. They used contrasts to their neighbour’s house cleanliness to convey this feeling of inadequacy. *“…when smoke is pouring into my home and realizes that neighbours’ houses are cleaner than mine, I feel that I am inferior…”* (*A 28-years-old participant, P8*).

#### Depression

Depression is another psychosocial health effect of pregnant women due to the suffocation of their house by smoke. The extreme condition of household air pollution leads to depression that mentioned by eight participants. For instance one of the study participants from highland area mentioned the scenario as follows: *“Yes, this is the main problem I have since it makes me feel uncomfortable, unhappy, and my daily activities may not go as planned when the air inside the house is contaminated”.* (*A 28 years-old participant, P5*).

### Perceived barriers to tackle household air pollution

#### Financial constraints

Fourteen participants perceived lack of money to construct separate kitchen, to buy improved cooking stove and develop biogas were the major barriers to tackle household air pollution. A 23-years-old participant from the highland area mentioned that lack of money to tackle household air pollution was the major barrier: *“…If I have the money, I’ll purchase a better cook stove like the “Lakech”. Look, you experienced the smoke here as well; this was caused by our current economic standard. Nothing else is a barrier.” (* *A 23-years-old participant, P1* *)*.

#### Spouse negligence

Some pregnant women also perceived that their partner’s negligence is another barrier to tackle the household air pollution. One of the pregnant women mentioned the situation as follow: “*My husband was informed about the problem, but he is unconcerned with household air pollution. He viewed my anxiety as amusement*.” (*A 38 years-old participant, P4*).

#### Autonomy

The perception of pregnant women as a problem to tackle HAP is the level of independence that women have at home. One participant from the lowland area gave the following explanation: *“… As you are aware, we reside in a remote location and are entirely dependent on the resources of our husband. Nothing can be done without my husband’s approval.”* (*A 28 years-old participant, P15*).

#### Lack of knowledge

A woman’s knowledge on how to prevent household air pollution exposure is perceived as a barrier to tackle household air pollution. Five study participants believed knowledge limitation is a barrier to mitigate household air pollution exposure. A 28- year’s old participant, who have tukel house said that: *“…here in rural area we used a conventional type of cookstove because, as you are aware, we live outside of an urban area. I am unsure whether better cookstoves exist or not…”* (*A 28-years-old participant, P9*).

### Perceived solutions to mitigate household air pollution

#### Open the door and window

Opening the door and window is an activity done when the house suffocated by polluted air. Eleven study participants said that opening the door and window when the house is snaked by smoke, allows fresh air into the house and the suffocated air to leave. For instance, one of the participants who live in the highland area mentioned: *“When the house is suffocated by smoke, I immediately open the window and door, and then out from the house until the suffocated air becomes refreshed…”* (*A 38-years-old participant, P4*).

#### Construct separate kitchen and increase ceiling height of house

This subcategory addresses an activity made on the design of the main house and on the kitchen to prevent HAP exposure. Some of the structural solutions suggested by the participant to mitigate household air pollution are constructing a separate kitchen from the main house and the height of the ceiling to be high. For instance, a participant who has a separate kitchen said: *“Having a separate kitchen from the main house is important to prevent air pollutant entrance to the main house.” (* *A 25-years-old participant, P14* *)*.

Another study participant stated the importance of having a high ceiling in the house as follows: *“….if the kitchen has a high ceiling height; it allows easy circulation of air in the house and refreshes the asphyxiated air.” (* *A 35-years-old participant, P2* *)*.

#### Use improved cookstove

Most women said that utilizing improved cookstove during preparation of their food could lessen the exposure of household air pollution. The following informant provided a description of using an improved cookstove to lessen exposure to indoor air pollution in homes: *“Yes, I did use a better cooking appliance, which helped me a lot from exposure to smoke and remove it from the house, freeing me from its effects.” (* *A 35-years-old participant, P12* *)*.

### Reduce workload

More than one third of the participants perceived that special care is needed during pregnancy to prevent HAP, reducing the exposure time in the kitchen, and workload of the pregnant women could prevent exposure of household air pollution One participant mentioned the following: *“…you know… more care is needed during pregnancy than free from pregnancy; therefore, I need to care when I sleep, when I work in the kitchen, and when I stay in the living room. In all my activities, I tried to reduce my exposure time from smoke, especially from charcoal smoke….” (* *A 20-years-old participant, P8* *)*.

Another 28 years old pregnant woman also adds on this as follows: *“Eee…. Yes, my older children do the most of the household duties. I had enough rest and stopped frequent trips to the kitchen to prepare our meals, which helped me to reduce my exposure to smoke pollution.” (* *A 20-years-old participant, P13* *)*.

## Discussion

This study explores pregnant women’s perceptions and experiences on the health impacts of household air pollution; barriers to tackle it and solutions to prevent it in rural Butajira, Ethiopia. It was interesting that air pollution was described by the participants as a smoke released from using biomass fuels as the main source of energy. However, some participants also felt that foul smell and household air pollution were synonymous, and that having a dirty house was also bad for health. This finding is consistent with the research done in Nepal [27]. The different in the definition of household air pollution among participants could be a result of the complexity of the problem and the participants’ varied perspectives. Therefore, a thorough definition of household air pollution should take into account its effects on human health, socioeconomic issues, and environmental repercussions [28]. These have implications for interventions that can tackle household air pollution.

The primary source of household air pollution, according to research participants, is the use of biomass fuel as an energy source. The results of this study are in line with the quantitative studies done in Cameroon [29]. The use of biomass fuels for cooking and heating accounts for a large portion of the indoor air pollution that occurs in houses and has a detrimental impact on both human health and the environment. Although there are cleaning alternatives, particularly for low-income households, fixing this issue requires a large financial commitment and a change in lifestyle.

This study identified the perceived health effects of household air pollution among pregnant women. The majority of the participant believed air pollution was an important cause of bad health, many saying that reductions would lead to benefits in their lives. They identified eye irritation and respiratory problems like coughing, sneezing and asthma were the major health problems caused by household air pollution exposure. This finding corresponds with previously published research [27, 30, 31]. Evidence also suggests air pollution can cause oxidative stress and inflammation in the smaller airways, leading to the exacerbation of asthma and chronic bronchitis, airway obstruction and decreased gas exchange. It can also undermine normal lung antimicrobial defence functions by interfering with the clearance and inactivation of bacteria in the lung tissue [32, 33]. Exposure to household air pollution can also harm the general health of the eyes and vision. Household air pollution can result in dry eye syndrome, watering and burning sensations, confused vision, and even glaucoma, the effects of which may be irreversible if exposed on a regular basis [34].

The health effects of household air pollution on the foetus were also clearly identified by the study participants, such as asphyxiated, reduce weight, abortion and hydrocephalus among foetus, similar to the quantitative study findings in different parts of the world [35, 36]. This may be due to household air pollutants which enter to the maternal blood, cross the placental barrier and have direct toxic effects on the foetus. Exposure to high levels of carbon monoxide, which binds to haemoglobin to form carboxy haemoglobin, reduces the capacity of the blood to carry oxygen to body tissues. Thus, a developing foetus can be deprived of adequate oxygen leading to intra-uterine growth retardation and risk of low birth weight and stillbirth [37]. The exposure to particulate matter can also result in decreased efficiency of trans-placental function, which can lead to consequent deterioration of foetal growth and development [38]. Inhaling household air pollutants can result in respiratory infections among foetus, which can cause breathing difficulties. Those health effects may also be caused by different contributing factors like nutrition and the health status of the mother. Therefore to make women more supportive further longitudinal study is needed in order for interventions to be designed and implemented in acceptable and effective ways.

In this study, participants expressed that household air pollution has significant psychosocial effect that can lead to inferiority, depression and various mental health issues. It was consistent with the study done in Nepal [27]. Respiration rate during pregnancy increases to support the growing foetus which actually increases maternal vulnerability to the harmful effects of air pollution. This air pollution could activate neuro-inflammatory pathways which lead to maternal depression and feeling of inferiority [39, 40]. Another justification for this may be people who live in homes with solid fuel are frequently mocked because of poverty and a lack of access to renewable energy sources. Feelings of infertility and humiliation may result from this, which has an impact on mental health.

The barriers to tackle household air pollution are complex and multifaceted, with financial constraints; spouse negligence, lack of full autonomy by the women and lack of knowledge were some of the major challenges mentioned by the study participants. This was also mentioned in previous research in Nepal, Rwanda and Kenya [27, 30, 31, 41], and possibly due to the lack of involvement of men in maternal healthcare services. It is known in many developing countries that men are the key decision-makers and chief providers of economic resources for their wives and their role is highly influential in women’s health care activities, especially in indoor health [42]. To overcome these barriers, it is necessary to implement targeted policies and programs that address these issues and empower women to make decision about energy use in their households, additionally, improving access to financing and promoting the use of renewable energy technologies can help reduce the financial burden of clean energy adoption.

## Limitation of the study

The ideas raised by the pregnant women should be considered as context-specific. Lack of extensive probing questions during data collection makes the finding somewhat limited. The distinctions in understanding are context-specific and cannot be generalized to other contexts. Despite these limitations the study still provides useful insights on the perceptions of pregnant women on health effects of household air pollution among the pregnant women.

## Conclusions

The health impacts of indoor air pollution on pregnant women are thoroughly explored in this study. In this study, we found that pregnant women’s perceptions of the health effects of household air pollution were contentious. The vast majority of the participants believed that eye irritation and respiratory issues like coughing and sneezing were the major health effects of HAP on the women, and reduce weight; abortion and hydrocephalus were the effects of HAP on the foetus. In addition to being highlighted as psychosocial health effects of household air pollution, depression and inferiority was mentioned. The pregnant women said that addressing home air pollution exposure was hampered by a lack of financial resources, spouse negligence and knowledge. The pregnant women saw the construction of separate kitchens, using improved cookstove, and home ventilation as the best solutions. The results of this study are crucial for developing effective intervention strategies to reduce household air pollution. In order to reduce exposure to household air pollution, it is crucial to educate women about ways to reduce it, improve existing home structures, and shorten pregnant women’s exposure times. It is necessary to conduct additional longitudinal study to investigate the health effect of household air pollution on the foetus health.

## Data Availability

The datasets used and/or analyzed during the current study will be available from the corresponding author on reasonable request.

## Abbreviations

HAP: Household Air Pollution
PhD: Doctor of Philosophy
SPH: School of Public Health
WHO: World Health Organization
